# Neuroprotection by α2-Adrenergic Receptor Stimulation after Excitotoxic Retinal Injury: A Study of the Total Population of Retinal Ganglion Cells and Their Distribution in the Chicken Retina

**DOI:** 10.1371/journal.pone.0161862

**Published:** 2016-09-09

**Authors:** Caridad Galindo-Romero, Mohammad Harun-Or-Rashid, Manuel Jiménez-López, Manuel Vidal-Sanz, Marta Agudo-Barriuso, Finn Hallböök

**Affiliations:** 1 Department of Neuroscience, Uppsala University, Box 593, 751 24 Uppsala, Sweden; 2 Instituto Murciano de Investigación Biosanitaria Hospital Virgen de la Arrixaca (IMIB-Virgen de la Arrixaca) and Departamento de Oftalmología, Facultad de Medicina, Universidad de Murcia, Murcia, Spain; University of Miami, UNITED STATES

## Abstract

We have studied the effect of α2-adrenergic receptor stimulation on the total excitotoxically injured chicken retinal ganglion cell population. N-methyl-D-aspartate (NMDA) was intraocularly injected at embryonic day 18 and Brn3a positive retinal ganglion cells (Brn3a+ RGCs) were counted in flat-mounted retinas using automated routines. The number and distribution of the Brn3a+ RGCs were analyzed in series of normal retinas from embryonic day 8 to post-hatch day 11 retinas and in retinas 7 or 14 days post NMDA lesion. The total number of Brn3a+ RGCs in the post-hatch retina was approximately 1.9x10^6^ with a density of approximately 9.2x10^3^ cells/mm^2^. The isodensity maps of normal retina showed that the density decreased with age as the retinal size increased. In contrast to previous studies, we did not find any specific region with increased RGC density, rather the Brn3a+ RGCs were homogeneously distributed over the central retina with decreasing density in the periphery and in the region of the pecten oculli. Injection of 5–10 μg NMDA caused 30–50% loss of Brn3a+ cells and the loss was more severe in the dorsal than in the ventral retina. Pretreatment with brimonidine reduced the loss of Brn3a+ cells both 7 and 14 days post lesion and the protective effect was higher in the dorsal than in the ventral retina. We conclude that α2-adrenergic receptor stimulation reduced the impact of the excitotoxic injury in chicken similarly to what has been shown in mammals. Furthermore, the data show that the RGCs are evenly distributed over in the retina, which challenges previous results that indicate the presence of specific high RGC-density regions of the chicken retina.

## Introduction

Excitotoxic injury has been used extensively to study cell death and proliferation in the retina. Treatment of the developing chicken retina with excitotoxins like N-methyl-D-aspartate (NMDA) induces cell type-, developmental stage- and regional-specific injuries [1–4]. The excitotoxic injury also produces a robust gliotic response by Müller cells with dedifferentiation, proliferation and formation of Müller cell-derived retinal progenitors [5]. Activation of α2-adrenergic receptor (α2-ADR) signaling reduces the adverse effects by different types of injury on retinal neurons. This has specifically been studied by analyzing rodent retinal ganglion cell (RGC) loss after injury [6–12]. The underlying mechanisms for the neuroprotection is not fully understood but is suggested to encompass modulation of excitotoxic signaling directly on RGCs, promotion of neurotrophic factor synthesis in the injured retina or attenuation of the gliotic response by the Müller cells and thus promotion of neuronal survival by maintenance of retinal homeostasis [6, 13–20]. We have recently shown that activation of α2-ADR on Müller cells modulates the injury-response by attenuating epidermal growth factor receptor- (EGFR) triggered extracellular signal-regulated kinase (ERK) signaling [18]. EGFR and ERK signaling has a central role in the regulation of the injury-response by Müller cells [21] and modulation of the injury-response by α2-ADR on Müller cells is likely to be part of the mechanism of α2-ADR agonists that promote neuronal survival. There are several α2-ADR agonists including xylazine, dexmedetomidine and brimonidine that have similar effects, but brimonidine, which is also used as a glaucoma drug [22], has been extensively studied in different retinal injury-models [23]. Because α2-ADR agonists have robust effects on Müller cells as studied in the chicken retina [18, 19] and since it was not known if α2-ADR agonists have similar neuroprotective effects in chicken as in mammals, we studied the effect of brimonidine on chicken RGC loss after an excitoxic lesion by NMDA.

We have used an automated method based on flat-mounted whole retina and immunostaining for the RGC-specific transcription factor Brn3a to study the total RGC population. Cells with immunoreactivity (IR) for Brn3a (Brn3a+ RGCs) were counted and isodensity maps were generated to visualize the topographical distribution of Brn3a+ RGCs. This method has successfully been used in mammalian species to analyze the RGC population after injury [23–25]. Brn3a is a member in the POU4f transcription factor family that is directly involved in the formation of RGCs [26, 27]. The expression is restricted to RGCs and it first appears in early differentiating chicken RGCs by embryonic day 5–6 (E5-6) [28, 29]. Flat-mount retinal dissection is a technique that allows studies of the whole retinal population [30, 31]. Previous studies have quantified the population of RGCs based on the number of optic nerve fibers or the number of cells in the ganglion cell layer with subtraction of non-RGCs [32–34].

The main objective of this work was to study the effects of brimonidine on the survival of injured chicken RGCs. An excitotoxic lesion was inflicted and the total population of RGCs was studied by automated counting of Brn3a+ RGCs in flat-mount retinas. A side-result from the flat-mount analysis showed the topographical distribution of the total RGC population in the chicken retina. We tested the effect of a single bolus intravitreal injection of brimonidine one hour before the lesion at E18 in the late chicken embryo and counted the RGCs 7 or 14 days later. The age was selected because we have previously seen that brimonidine and NMDA give a robust retinal response at that age with activation of Müller cell gliosis. In order to study if the RGC population changes during the experimental period, a series of normal chicken retina from E8 to post-hatch day 11 (P11) was analyzed. The result showed that pretreatment with brimonidine significantly reduced RGC loss as seen both 7 and 14 days post lesion (dpl). The excitotoxic lesion was more severe in the dorsal than in the ventral retina. A similar regional difference was also seen in the effect of brimonidine on RGC survival.

## Materials and Methods

### Animals

Fertilized White Leghorn eggs from a local breed were obtained from OVA Produktion AB (Västerås, Sweden) and incubated at 38°C in a humidified incubator (Maino, Naples, Italy). After hatching, chicks were marked with a numbered plastic ring in the leg and moved to a poultry farm at the National Veterinarian Institute, Uppsala, Sweden. The chicken were fed *ad libitum* and kept under standard conditions according to the legislation of the Swedish board of agriculture. Animal experiments were performed according to the guidelines given by the Association for Research in Vision and Ophthalmology and were scrutinized and approved by the local animal ethics committee: Uppsala försöksdjursetisk nämnd.

### Intraocular injections

Animals were divided in the following experimental groups:

1. The normality of Brn3a+ RGCs in control chicken retinas was analyzed in flat-mounts retinas of: E8, E9, E10, E12, E14, E18, E20; and post-natal days (P): P4 and P11 (n = 4/group).
2. To determine the dose of NMDA (M3262, Sigma-Aldrich, St. Louis, MO, USA), a pilot experiment was carried out. A single intraocular injection of 10 μl was made in E18 chick embryos, with three different doses of NMDA, 10, 50 and 100 μg (n = 3/group). Eggs were opened at the blunt end and a small hole was made in the eggshell and chorioallantoic membranes. The head was pulled with a bent glass rod and intraocular injection was done through the amniotic membranes in the right eye with a Hamilton syringe with a 27-gauge needle equipped with a rubber stop to position the tip of the needle in the center of the eye. After the injection, the egg was closed with a porous adhesive tape and kept in the incubator. Retinas were analyzed as cross-sections at time-points: 7 days (10 and 50 μg NMDA) and 14 days post-lesion (dpl) (10, 50 and 100 μg NMDA). The number of Brn3a+ RGCs in the GCL of 3 sections per retina were counted (n = 3 retinas per group). The result showed that 10μg NMDA gave a clear lesion and we therefore choose 5 and 10 μg NMDA for the main experiment.
3. Brimonidine intravitreally pretreated (80 μg brimonidine in 10 μl sterile saline, 0.15 M NaCl, UK 14,304 tartrate; Tocris Bioscience) NMDA-injured (5 or 10 μg NMDA in 10 μl) retinas were analyzed as flat-mounts at 7 and 14 dpl (n = 4/group). Pretreatment was one hour before NMDA injection. A saline-only-treatment group with two saline injections, one hour apart, was control for injection effect. All treatment groups were analyzed as flat-mounts at 7 and 14 dpl as well (n = 4 retinas/group).

### Retinal dissection

Both chick embryos and post-natal chickens were decapitated and eyes were enucleated and fixed in 4% paraformaldehyde (PFA) for 1 hour and kept in 0.1 M phosphate buffer saline (PBS) at 4°C. For flat-mount dissection, eyes were covered with cold Mg2^+^ and Ca2^+^ free PBS to facilitate the detachment of the vitreous body from the retina. First, the cornea was removed with scissors. Both limbus and iris were separated with forceps, and the sclera was separated from the choroid by making radial cuts from the anterior to the posterior pole. With the sclera only attached to the pecten oculli, the retina could be cut loose in one piece. The lens was removed and the retina was dissected following the protocol previously described in rats and mice [35, 36]. Briefly, four deep cuts were done from the anterior to the posterior pole, leaving the pecten oculli as a reference point, in the ventro-nasal part of the retina. The vitreous body was removed with forceps, from the center to the periphery of the retina. Finally, small cuts were done in each quadrant, to allow the retina to flatten completely. Flat-mounted retinas were fixed again for 1 hour in 4% PFA and kept in PBS. Quadrants were denoted: dorso-temporal (D-T), dorso-nasal (D-N), ventro-temporal (V-T), and ventro-nasal (V-N) with pecten oculli in the V-N quadrant.

For cross-sectioned retinas, the cornea was removed, the eye cup was incubated in 30% sucrose for 24 hours at 4°C. Eye cups were then embedded in Neg-50TM (Thermo Scientific) and kept at -70°C until cryo-sectioning. Retinal sections parallel to the center of the lens and through the optic nerve head were collected and frozen at -20°C.

### Immunohistochemistry

Brn3a whole-mount immunohistochemistry was carried out following the protocol used for rat and mouse retinas [24, 25]. Briefly, after fixation, retinas were permeated with PBS 0,5%Triton X-100 by freezing during 15 min at– 70°C in 24 well-plate, rinsed in PBS 0,5% Triton and incubated overnight at 4°C with primary antibody in blocking buffer (PBS 2%, bovine serum albumin, 2% Triton). After the incubation, retinas were washed four times in PBS 0,5% Triton and incubated 2 hours at room temperature with the secondary antibody in PBS with 2% Triton. Finally, retinas were washed in PBS and mounted with ProLong Gold Antifade mountant (Molecular Probes, Thermo Fischer Scientific Cat# P10144). Immunohistochemistry on sections was carried out as described [37–39].

Antibodies:

1. Mouse Anti-Brn3a (Millipore Cat# MAB1585 RRID:AB_94166, dilution 1:200)
2. Rabbit anti-rhodopsin (Cosmo Bio Co Cat# LSL-LB-5555 RRID:AB_605318, dilution 1:500)
3. Donkey anti-mouse Alexa Fluor 488 IgG (H+L) (Thermo Fisher Scientific Cat# A-21202 RRID:AB_2535788, dilution 1:1000) and
4. Donkey anti-rabbit Alexa Fluor 568 IgG (H+L) (Thermo Fisher Scientific Cat# A10042 RRID:AB_11180183, dilution 1:1000)

### Image acquisition

Brn3a IR fluorescence was captured focusing on the GCL at 10x magnification with a fluorescence microscope (Zeiss AxioImager 2, Zeiss microscopes, Intn.) using Alexa Fluor 488 channel and an exposure time of 1000 ms for each frame. The image capture was in tile acquisition mode with an overlap of 5% between frames. The resulting image reconstruction was exporting as .tiff file for further analysis. Because of the big size of our samples, the image reconstruction of each retina was carried out in four parts, following the four quadrants of each retina (D-T, D-N, V-N and V-T).

### Whole-mount retinal analyses

#### Automatic quantification of the total number of Brn3a positive RGCs

Every quadrant of the retina was analyzed separately. The automated Brn3a+ cell counting routines for Image-Pro Plus (Image-Pro Plus, IPP 5.1 for Windows; Media Cybernetics, Silver Spring, MD) for rat [24, 40, 41] and mouse retinas [25, 42] were adapted to the analysis of chicken retinas. The chicken retinas are larger and contain in the order of 50 times more RGCs than a rat retina. Images were orderly selected from the reconstructed quadrant and converted to 16-bit gray scale, followed by the application of the higauss 7x7 enhancement filter. The resulting images were then filtered through a large spectral filter: Edge+, which extract positive edges from the dark background. Potential cell clusters were separated by the IPP watershed split morphologic filter. Cells were counted within predetermined parameters to exclude objects that are too large and too small to be the RGCs nuclei. Finally, data of each count were displayed and exported to a spreadsheet where they were filed and saved for further analysis. The total number of Brn3a^+^RGCs was obtained as the sum of the four quadrants of each retina.

#### Validation of automatic counting

The Brn3a staining and automatic counting was validated by manual counting. Evenly distributed sample images (18 images; 4 images from each quadrant and 2 from the central retina) from the GCL were selected from an E18 flat-mount retina. Brn3a+ and DAPI+ nuclei were counted to determine the fraction of Brn3a cells to DAPI+ cells in the GCL and cell density.

#### Spatial distribution of Brn3a positive RGCs

Detailed spatial distribution of Brn3a+ RGCs over the entire retinas was obtained through quadrant analysis with areas of study of 0.0073 mm^2^, and demonstrated with isodensity maps constructed as previously described [35, 36, 43].

#### Measurement of retinal area

Retinal area was measured by manually drawing the retinal contour on each quadrant using IPP software, total area of each retina was obtained as the sum of the four quadrants measurements.

### Statistical analysis

To compare values from different quadrants of the retina and also to compare retinas from different groups, we used the ANOVA test and Tukey posthoc test (SigmaStat^®^ for Windows^™^ Version 3.11; Systat Software, Inc., Richmond, CA).

## Results

### Brn3a positive RGCs in normal retinas from embryonic day 8 to post-hatch day 11

**Fig 1:**
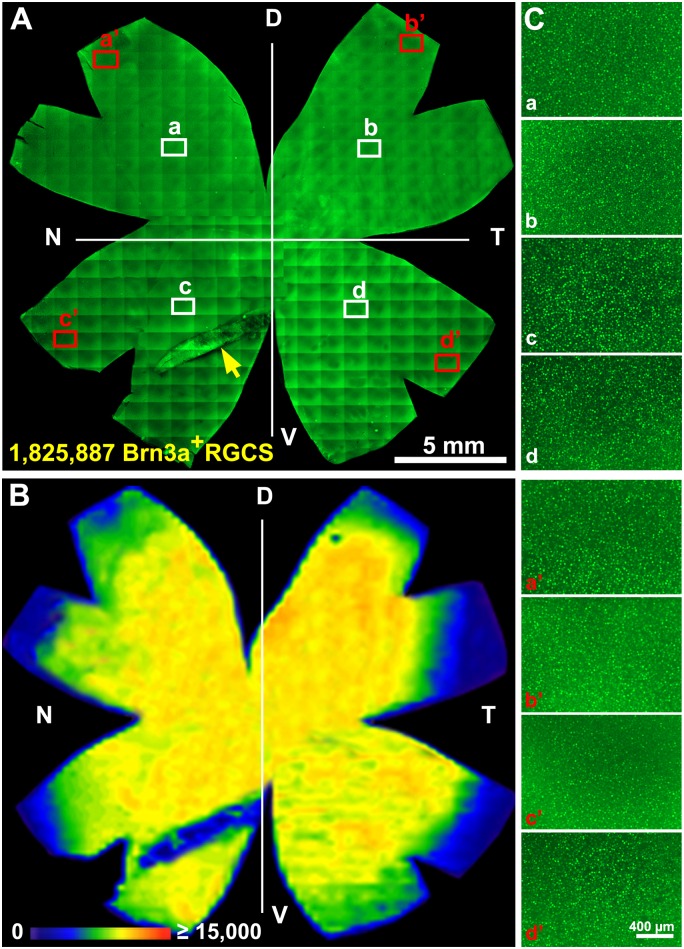
Distribution of Brn3a+ RGCs in the E18 retina. Flat-mounted E18 retina immunostained for Brn3a RGCs. (A) Reconstruction of an entire retina with tiled 100X fluorescence micrographs indicating the retinal quadrants. Reconstruction was used for the automated RGC-counting and the total number of Brn3a+ RGCs is indicated. Yellow arrow indicates the pecten oculi. (B) Isodensity map of Brn3a+ RGCs in retina shown in A. The map is a filled contour plot generated by assigning to each individual frame a color code according to its RGC density, within a scale ranging from 0 to 2,000 (purple) to 15,000 or more (red) RGCs/mm^2^ (bottom left). (C) Representative micrographs from the four quadrants of the retina shown in A with Brn3a+ RGCs. White and red rectangles in (A) indicate the location of the depicted areas (a-d, a’-d’). E; embryonic day, D; dorsal, T; temporal, N; nasal, V; ventral.

**Fig 2:**
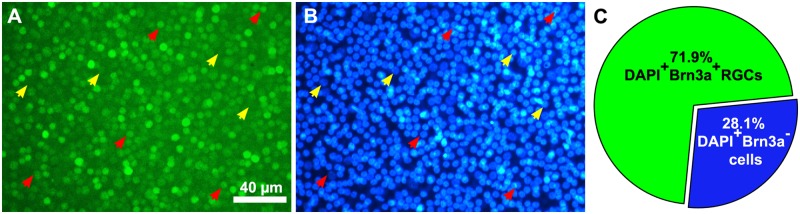
The fraction of Brn3a+ cells in the ganglion cell layer of E18 retina. Representative fluorescence micrograph image sample of the ganglion cell layer (GCL) from E18 chicken retina with (A) Brn3a immunoreactivity and (B) DAPI nuclear staining. Red arrows indicate Brn3a, DAPI double positive cells. Yellow arrows indicate DAPI+, Brn3a negative cells. (C) Fraction of Brn3a+ cells of DAPI+ cells in the GCL. The number of Brn3a+ cells and DAPI+ cells was counted manually in 18 evenly distributed sample areas of an E18 flat-mount retina. Cell counts are listed in S1 Table in supporting file.

Whole-mount retina reconstruction showed that Brn3a+ cells were distributed over the entire retina, except for the region of the pecten oculli (Fig 1A). Fewer cells were seen in the peripheral rim of the retina. We compared Brn3a+ RGCs in total retina, in the dorsal and ventral halves and in the four retinal quadrants: dorso-nasal (D-N), dorso-temporal (D-T), ventro-nasal (V-N) and ventro-temporal (V-T). Pecten oculli is located in the V-N quadrant. The E18 retina depicted in Fig 1A contained 1.82x10^6^ Brn3a+ cells. The data were used to generate cell density maps to display the topographic distribution of Brn3a+ RGCs throughout the retina. Fig 1B shows an isodensity map of the E18 retina. The average Brn3a+ RGC density was 10.1x10^3^±1.2x10^3^ cells/mm^2^. Micrographs from the central and peripheral part of the four quadrants show the resolution of the Brn3a+ RGCs in the flat-mount (Fig 1Ca–1Cd and 1Ca’–1Cd’). DAPI was used to stain nuclei and we manually counted DAPI stained nuclei and Brn3a, DAPI double positive nuclei in 18 sample areas (Fig 2A and 2B). The manually determined average density of Brn3a+ cells in the sample areas was 11.0x10^3^±2.8x10^3^ cells/mm^2^. 72±10% of all DAPI+ nuclei were Brn3a+ (Fig 2C).

**Fig 3:**
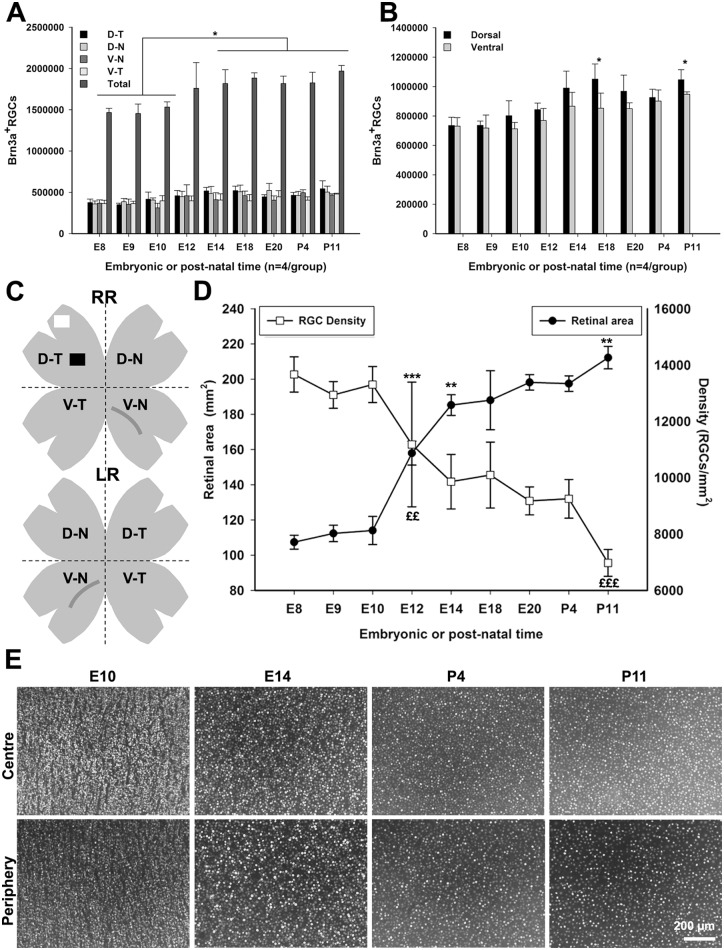
Brn3a+ RGCs in normal embryonic and post-hatch control retinas. Number and density of Brn3a+ RGCs in normal embryonic (E8—E20) and post-hatch (P4, P11) retinas. Brn3a+ RGCs were counted using automated cell counting in four retinal quadrants of flat-mount retina. (A) Retinal quadrants, (B) retinal halves (dorsal and ventral) and (A) whole retina from the different ages were compared. (C) Schematic diagram of flat-mount right (RR) and left (LR) retina with quadrants depicted. (D) The total area of flat-mounted retina plotted against the Brn3a+ RGC density (total Brn3a+ RGCs/retinal area) for each age. (E) Representative fluorescence micrographs of Brn3a+ cells in central and peripheral E10, E14, P4 and P11 retinas. One way ANOVA and Tukey’s post-hoc test, * = p<0.05, ** = p< 0.01, *** = p<0.001; £ = p<0.05; ££ = p< 0.01 and £££ = p<0.001, for retinal mean density. Cell counts are listed in Table 1. D-T; dorso-temporal, D-N; dorso-nasal, V-N; ventro-nasal, V-T; ventro-temporal.

**Fig 4:**
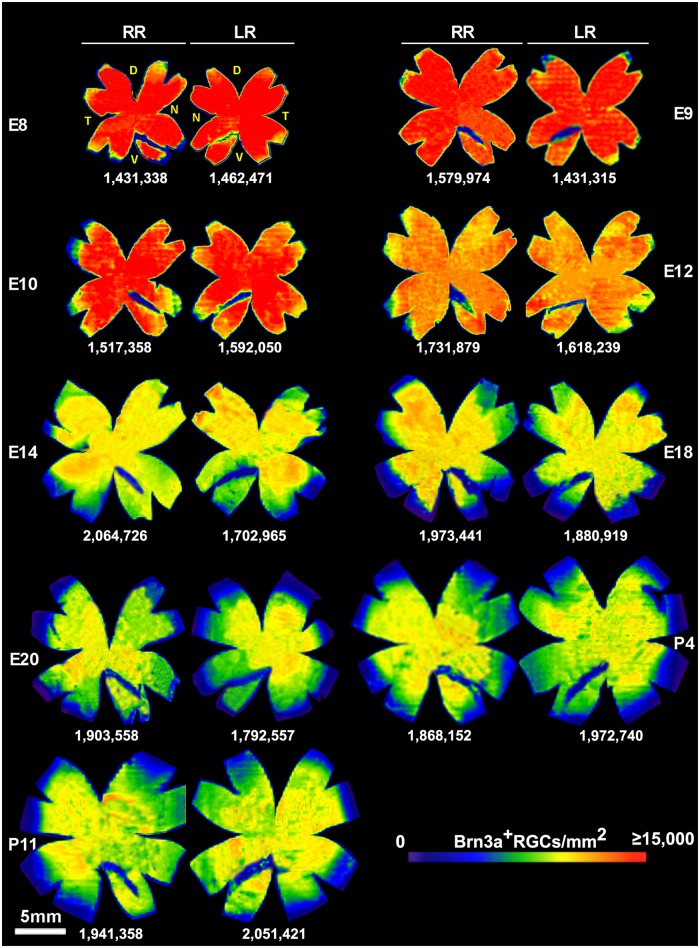
Topographic distribution of Brn3a+ RGCs in normal embryonic and post-natal control retinas. Isodensity maps showing the topographic distribution of Brn3a+ RGCs in normal E8—E20 and P4, P11 control retinas. A representative right (RR) and left (LR) retinas is shown for each age. The total number of Brn3a^+^RGCs in each retina is indicated. D; dorsal, T; temporal, N; nasal, V; ventral.

The number of Brn3a+ RGC in retinas from E8 to P11 is plotted per quadrant (Fig 3A and 3C. Data are listed in S1 Table), per half retina (Fig 3B) and per whole retina (Fig 3A). Data are listed in Table 1. There was more Brn3a+ RGC in the dorsal half than in the ventral (Fig 3B), however, if the number of RGCs equivalent to the area of the RGC-free region occupied by the pecten oculli in the V-N quadrant was added to the total number of RGC in the ventral half, the difference disappeared (data not shown). The area of the retinas and the Brn3a+ RGC cell density for retinas from E8 to P11 was determined (Fig 3D, Table 1). The number of Brn3a+ RGCs in E8-E10 retinas was approximately 1.5x10^6^ cells and in E12-P11 approximately 1.8x10^6^ cells per retina. Two different age-groups were seen (p<0.05). Micrographs from the center and periphery of E10, E14, P4 and P11 flat-mounted retina are shown in Fig 3E. Isodensity maps of the Brn3a+ RGC density displayed the spatial distribution of RGCs and size of the flat-mounted retinas at the selected ages (Fig 4). We did not find any specific regions with increased RGC density. The Brn3a+ RGC density decreased with age as shown by the colder colors of the isodensity map (se also Fig 3D).

**Table 1:** Number, retinal area and Brn3a+ RGC density in embryonic and post-natal control retinas.

		Retinal halves		Total
Age		Dorsal	Ventral	
E8	Brn3a^+^RGCs	735,200±56,243	731,097±57,397	1,466,296±50,976
	Area^1^	50.1±1.1	57.3±2.8	107.4±4.0
	Density^2^	14,667±1,064	12,813±1,430	13,667±626
E9	Brn3a^+^RGCs	736,799±28,959	718,365±87,927	1,455,164±114,760
	Area	54.2±2.0	58.1±6.4	112.3±4.7
	Density	13,603±929	12,354±183	12,940±475
E10	Brn3a^+^RGCs	824,075±93,743	709,900±36,253	1,533,975±60,863
	Area	59.3±7.5	54.7±2.6	114.0±8.0
	Density	13,535±679	13,030±831	13,308±638
E12	Brn3a^+^RGCs	905,474±129,442	854,901±182,913	1,760,375±310,964
	Area	77.7±2.4	80,2±5.5	158±6.8***
	Density	11,682±1,965	10,705±2,471	11,181±2,215*
E14	Brn3a^+^RGCs	1,000,897±96,370	816,898±125,385	1,817,795±168,493*
	Area	101.0±6.8	84.3±7,5	185.3±5.9
	Density	9,769±1,597	10,029±231	9,861±968
E18	Brn3a^+^RGCs	1,027,698±95,771	856,371±83,998	1,884,069±63,689
	Area	97±10.8	91±7.2	188.0±16.8
	Density	10,683±1,483	9,480±1,499*	10,096±1,170
E20	Brn3a^+^RGCs	968,301±108,897	849,850±39,637	1,818,151±89,297
	Area	107.5±2.1	90.7±2.3	198.2±4.4
	Density	9,004±976	9,386±680	9,178±496
P4	Brn3a^+^RGCs	925,607±56,449	900,944±75,782	1,826,550±128,337*
	Area	97.5±2.3	100±3.5	197.5±4.4
	Density	9,488±382	9,030±1,014	9,252±688
P11	Brn3a^+^RGCs	1,046,562±68,327	947,436±16,470	1,993,998±55,187*
	Area	108±5	104±5	212.2±6.4**
	Density	9,692±1,057	9,133±451	9,404±450**

Numbers are mean±SD and are plotted in Fig 3A and 3B, ANOVA

* = p<0,05;

** = p< 0,01 and

*** = p<0,001. 1) Area (mm^2^), 2) Density (Brn3a^+^RGCs/mm^2^).

### Determination of NMDA-dose for the excitotoxic injury

**Fig 5:**
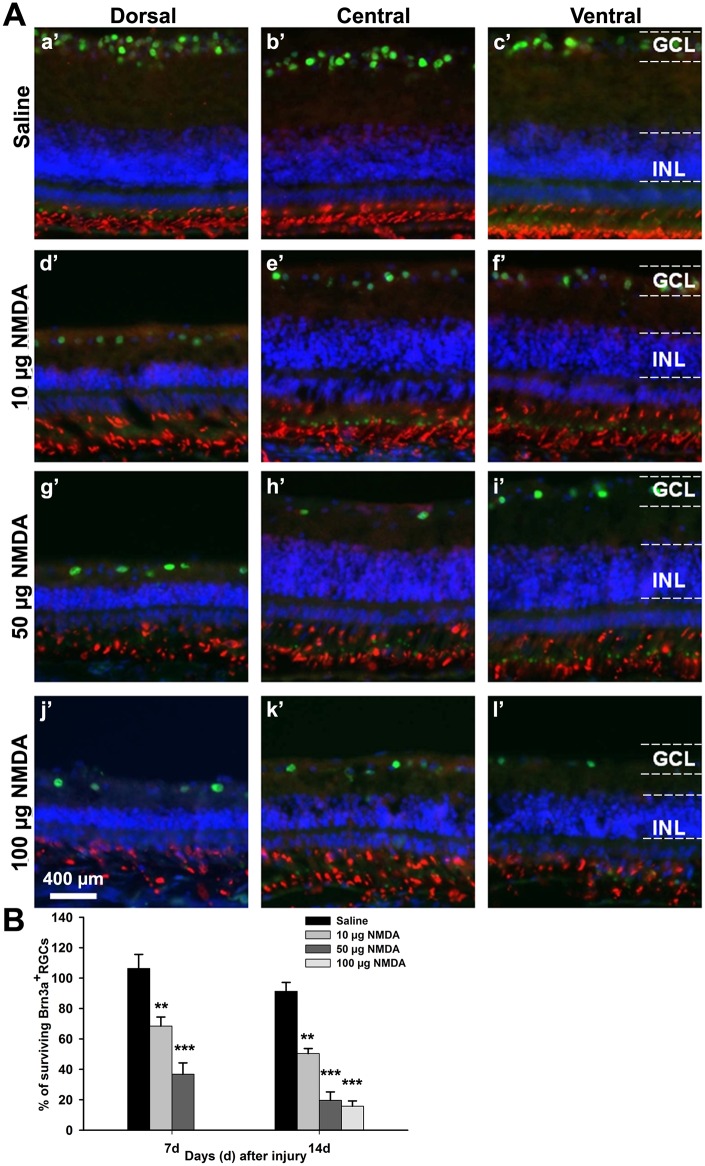
Dose-dependent excitotoxic injury of Brn3a+ RGCs. Effect of intraocular injection of different amounts of NMDA on Brn3a+ RGC survival. (A) Fluorescence micrographs of cross sections of P11 retina 14 days after injection of saline (a’-c’), 10 μg of NMDA (d’-f’), 50 μg of NMDA (g’-i’) and 100 μg of NMDA (j’-l’). Brn3a (green), rhodopsin immunoreactivity (red) and DAPI (blue). (B) Bar graph showing survival of Brn3a+ RGCs at 7 and 14 days post-lesion. 100 μg NMDA was only analyzed 14 dpl. Cell-counts were on cross-sections and are shown as percentage of Brn3a+ cells in normal control retina (100%, not shown). One way ANOVA, Tukey test post-hoc test, n = 4, ** = p<0.01, *** = p< 0.001. GCL; ganglion cell layer, INL; inner nuclear layer.

Previous studies have used high dose of NMDA (>100 μg NMDA) in order to induce total cell loss or to inflict a robust glial injury-response [18, 19]. In this study we sought to make a less severe injury, which would allow and be used to assess the neuroprotective effect of brimonidine. We tested in a pilot experiment three doses of NMDA; 10, 50 and 100 μg and compared to saline. Cross-sections of the retinas were analyzed at 7 and 14 dpl (except for 100 μg, time-point 7 days) by immunohistochemistry for Brn3a and the photoreceptor marker rhodopsin. The results from the 14 dpl showed that all three NMDA doses induced a clear reduction of both the GCL and the inner nuclear layer thickness. The ventral, central and dorsal portions of the retina showed partially different results with a more severe reduction of the thickness in the dorsal portion of the retina (Fig 5A). The rhodopsin+ cells (red) appeared disorganized in the lesioned retina but were not lost (Fig 5A). The number of Brn3a+ RGC in the GCL was clearly reduced compared to saline. 50 μg NMDA induced a more than 50% Brn3a+ RGC (green) loss already at 7 days that increased to more than 75% loss at 14 dpl. 100 μg NMDA induced more than 80% loss at 14 dpl (Fig 5B). Ten μg of NMDA induced a milder injury but the loss of RGCs was still approximately 50%. Based on these results we decided to use the doses 5 and 10 μg NMDA for the present study.

### Pretreatment with brimonidine reduced the adverse effect of NMDA on Brn3a+ RGC survival

**Fig 6:**
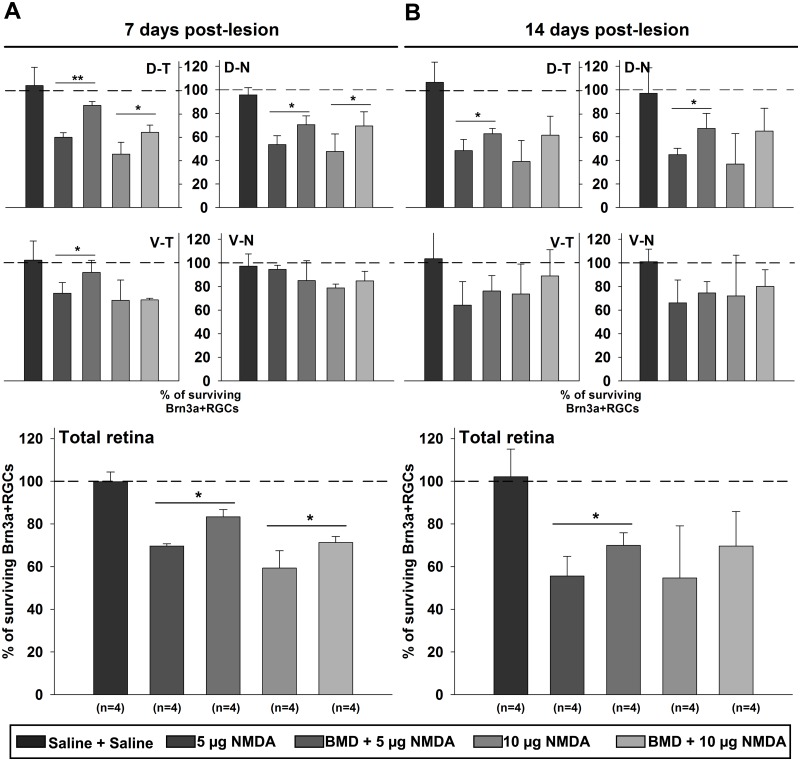
Effect of brimonidine pretreatment on excitotoxically injured Brn3a+ RGCs. Bar graphs showing survival of Brn3a+ RGC in retinas after NMDA injury pretreated with saline or brimonidine (BMD). Brn3a+ RGCs were counted using the automated cell counting in four retinal quadrants of flat-mount retina and survival is shown as percentage of the number of Brn3a+ cells in normal control retina (100%). Analysis of Brn3a+ cell survival at (A) 7 days and (B) 14 days post-lesion for each retinal quadrant and for the total retina. Cell counts are listed in Tables 2 and 3. One way ANOVA, Tukey’s post-hoc test, n = 4, * = p<0.05, ** = p< 0.01. D-T; dorso-temporal, D-N; dorso-nasal, V-N; ventro-nasal, V-T; ventro-temporal.

Pretreatment by injection of brimonidine or saline was done 1 hour prior to injection of NMDA or saline into the right eye of E18 chickens *in ovo*. The study had five groups that were compared to the normal control retina. One control group received two intraocular injections of saline. The experimental groups either received injections of saline followed by injections of 5 or 10 μg NMDA or injections with brimonidine followed by 5 or 10 μg NMDA. The groups were taken 7 or 14 dpl (at P4 and P11) and analyzed by automatic cell counting of flat-mount retina stained for Brn3a. The cell counts were plotted in bar graphs as percent of the number of RGCs in normal control retina, which was denoted 100% (Fig 6). Cell count-data from 7 and 14 dpl are listed in Tables 2 and 3. The number of Brn3a+ RGCs in 7 dpl eyes that received two saline injections was similar to normal P4 control retina (1.83x10^6^±1.3x10^4^ and 1.88x 10^6^±1.1x10^4^, respectively), indicating that the double intraocular injections alone did not produce any Brn3a+ RGC loss (Fig 6, S1 and S2 Figs).

**Table 2:** Number, retinal area and Brn3a^+^RGC density in experimental flat-mount retinas 7 days post-lesion.

7 days post-lesion						
		Retinal quadrants				Total retina
Groups		D-T	D-N	V-N	V-T	
Saline+Saline	Brn3a^+^RGCs	491,963±60,758	491,764±46,147	479,948±65,055	413,467.3±39,197	1,877,143±110,121
	Area^1^	49.1±3.0	55.1±5.3	46.0±10.6	42.6±4.9	192.8±12.1
	Density^2^	10,092±1,804	8,962±856	10,633±1,388	9,773±1,261	9,775±982
5 μg NMDA	Brn3a^+^RGCs	281,689±18,609^££^***	264,165±36,836^£^***	340,236±41,236*	393,028±14,389	1,279,118±19,348***
	Area	46.1±4.8	47.3±3.1	47.0±4.1	46.2±3.4	186.6±10.9
	Density	6,142±537	5,576±524	7,317±1,423	8,556±817	6,873±392
BMD+5 μg NMDA	Brn3a^+^RGCs	409,250±15,250	347,520±36,470*	420,786±47,222	353,280±70,488	1,530,835±62,158***
	Area	52.1±2.8	53.0±1.4	47.3±4.2	50.1±4.2	202.5±5.5
	Density	7,876±523	6,566±768	8,978±1,563	7,094±1,494	7,561±292
10 μg NMDA	Brn3a^+^RGCs	214,347±47,025^£^***	234,772±73,527^£^***	312,546±78,655**	327,515±13,586*	1,089,179±149,658***
	Area	44.6±7.4	49.4±6.3	45.5±4.9	43.0±5.6	182.5±14.7
	Density	4,792±641	4,798±1,506	6,794±1,103	7,695±792	5,963±600
BMD+10 μg NMDA	Brn3a^+^RGCs	301,170±29,411***	341,793±59,728*	314,056±6,175*	352,751±33,045	1,309,770±50,624***
	Area	47.7±4.3	49.6±7.1	46.0±6.0	42.7±6.6	186.0±13.6
	Density	6,322±475	7,102±2,253	6,929±1,067	8,322±640	7,073±663

Numbers are mean±SD, ANOVA

* = p<0.05;

** = p< 0.01 and

*** = p<0.001 for comparisons between the control (saline) and different treatment groups of the same quadrants.

^£^ = p<0.05;

^££^ = p< 0.01 and

^£££^ = p<0.001 for comparisons between quadrants (DT:VT, DN:VN) from the same group. 1) Area (mm^2^), 2) Density (Brn3a^+^RGCs/mm^2^). BMD; brimonidine, D-T; dorso-temporal, D-N; dorso-nasal, V-N; ventro-nasal, V-T; ventro-temporal.

**Table 3:** Number, retinal area and Brn3a^+^RGC density in experimental flat-mount retinas 14 days post-lesion.

14 days post-lesion						
		Retinal quadrants				Total retina
Groups		D-T	D-N	V-N	V-T	
Saline+Saline	Brn3a^+^RGCs	549,654±88,342	503,303±112,513	469,456±100,840	488,310±51,787	2,010,722±224,395
	Area^1^	57.6±3.9	45.7±24.2	53.4±9.6	51.1±7.0	207.8±23.9
	Density^2^	9,573±1,702	20,861±24,369	8,949±2,045	9,710±1,788	9,702±676
5 μg NMDA	Brn3a^+^RGCs	250,144±48,427***	232,656±28,731**	290,871±90,531*	320,075±93,485**	1,093,745±180,964**
	Area	62.4±3.7	57.5±2.5	56.1±2.2	49.8±3.4	225.9±8.3
	Density	4,016±836	4,053±565	5,173±1,584	6,505±2,150	4,855±873
BMD+5 μg NMDA	Brn3a^+^RGCs	323,804±23,308**	348,535±66,498*	345,368±59,347	359,733±47,315**	1,377,440±115,707*
	Area	55.5±2.6	55.2±2.9	49.8±4.3	49.4±3.3	209.9±5.6
	Density	5,862±718	6,289±1,016	6,898±556	7,359±1,430	6,560±466
10 μg NMDA	Brn3a^+^RGCs	202,017±91,832***	191,745±133,994***	333,852±115,279	348,262±166,488	1,075,876±480,92***
	Area	63.8±4.4	50.5±8.2	52.3±4.5	53.0±4.8	219.6±9.4
	Density	3,196±1,601	4,000±2,894	6,473±2,580	6,417±2,547	4,928±2,323
BMD+10 μg NMDA	Brn3a^+^RGCs	317,054±83,964**	336,755±100,538*	403,435±100,458	386,933±68,458*	1,444,177±271,089***
	Area	58.8±8.2	60.2±4.2	53.1±4.8	51.2±5.2	223.2±7.6
	Density	5,464±1,478	5,668±1,919	7,640±1,975	7,655±1,832	6,482±1,269

Numbers are mean±SD, ANOVA

* = p<0.05;

** = p< 0.01 and

*** = p<0.001 for comparisons between the control (saline) and different treatment groups of the same quadrants. 1) Area (mm^2^), 2) Density (Brn3a^+^RGCs/mm^2^). BMD; brimonidine, D-T; dorso-temporal, D-N; dorso-nasal, V-N; ventro-nasal, V-T; ventro-temporal.

A clear loss of Brn3a+ RGCs was seen in retina injected with 5 or 10 μg NMDA at 7 dpl (Fig 6A and S1 Fig), and it was more severe at 14 dpl (Fig 6B and S2 Fig). Brimonidine pretreatment reduced the RGC loss in NMDA-injured retinas compared to the saline, as seen at 7 and 14 dpl (Fig 6A and 6B, S1 and S2 Figs). In retinas injected with 5 or 10 μg NMDA a significant decrease of Brn3a+ RGCs was seen in both dorsal quadrants and in the V-N quadrant but not in the V-N one (Fig 6, Tables 2 and 3).

For the 7 dpl time point, 69.6±1.1% and 59.3±8.1% of the control Brn3a+ RGCs remained in the 5 and 10 μg NMDA lesion retinas, respectively. Brimonidine pretreated retinas had significantly more Brn3a+ RGCs in all but the V-N quadrant. The same results were seen for both doses of NMDA. When the total retina was compared, 83.3±3.4% and 71.3±2.8% Brn3a+ RGCs remained after brimonidine treatment with 5 or 10 μg NMDA, respectively (Fig 6A). The brimonidine treatment increased the number significantly.

The number of Brn3a+ RGCs 14 dpl in P11 control and saline retinas was 1.96 x 10^6^±6.7x10^4^ and 2.01x10^6^±2.2x10^4^, respectively. In the experimental retinas, all retinal quadrants were affected by the injection of NMDA with both doses (Fig 6B). The brimonidine pretreated 5 μg NMDA lesion-group had a significant higher number of Brn3a+ RGCs in both dorsal quadrants than the saline pretreated NMDA-lesion group. The lesion, as well as the protection, was smaller in the ventral quadrants. The percent surviving Brn3a+ RGCs with 5 or 10 μg of NMDA lesion were 55.6±9.2% and 54.6±24.2%, respectively, and in the brimonidine-treated retinas, 70.0±5.9% and 69.7±16.1%, respectively (Fig 6B).

**Fig 7:**
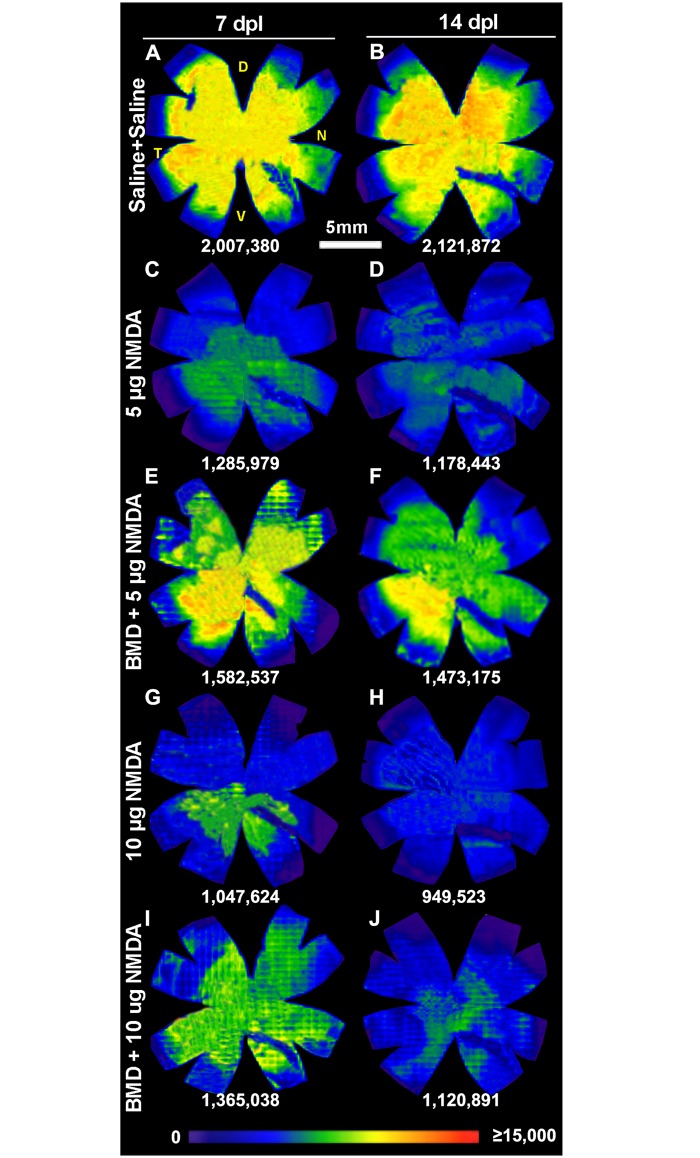
Topographic distribution of Brn3a+ RGCs in excitotoxically injured retina after brimonidine pretreatment. Isodensity maps showing the topographic distribution of Brn3a+ RGCs in a representative retina after NMDA injury pretreated with saline or brimonidine (BMD) at 7 days (A,C,E,G,I) and 14 days (B,D,F,H,J) post lesion. Experimental groups were: (A, B) Injections of saline + saline, (C, D) saline + 5 μg NMDA, (E, F) brimonidine + 5 μg of NMDA, (G, H) saline + 10 μg of NMDA, and (I, J) brimonidine + 10 μg of NMDA. The total number of Brn3a^+^RGCs of each retina is indicated for each map. BMD; brimonidine, D; dorsal, N; nasal, V; ventral, T; temporal.

The topographic distribution of the Brn3a+ RGCs displayed as isodensity maps, confirmed the pattern indicated by the cell count (Fig 7). The distribution of Brn3a+ RGCs was similar in saline retinas and control retinas. The isodensity maps showed that the NMDA injury mainly affected the dorsal quadrants. The Brn3a+ RGC density was lower in the dorsal than in the ventral quadrants and the effect of brimonidine pretreatment was mainly seen in the dorsal quadrants that were more severely affected by NMDA.

## Discussion

In this work we have studied the RGC population in normal and excitotoxically injured chicken retina after pretreatment with the α2-ADR agonist brimonidine. The primary objective was to study the effect of α2-ADR agonists in the chicken retina but the study also gave as a side-result: the distribution of the complete retinal ganglion cell population in the chicken retina. We used the RGC-specific transcription factor Brn3a to label flat-mounted retinas that were analyzed using automated cell counting. This method generated RGC isodensity maps, which displayed the regional distribution of RGC in normal, injured and neuroprotected retinas. The result showed that excitotoxic injury by intraocular injection of NMDA produced an injury with RGC loss that was more severe in the dorsal than in the ventral retina and brimonidine-pretreatment significantly reduced the loss of RGCs.

NMDA was intraocularly injected at E18 and retinas were analyzed at 7 and 14 dpl, corresponding to P4 and P11, respectively. We injected 5 or 10 μg NMDA for this long-term experiment. The dose was 10–20 times lower than what has often been used for short-term experiments. The loss of Brn3a+ RGCs was 31% and 41% at 7 dpl and approximately 45% at 14 dpl, depending on the NMDA dose. Injection of 100 μg NMDA, increased the cell loss to more than 80% at 14 dpl (Fig 5B), showing that the loss of RGCs and thus the severity of the injury was dose dependent. The dose-effect was similar to data presented in previous work [1, 4]. The selected doses of 5 and 10 μg NMDA for the experimental groups produced a robust and reproducible injury but it was not so severe than that the effect of brimonidine could be discerned. The brimonidine treatment reduced the cell loss to 17% and 29% at 7 dpl and approximately 30% at 14 dpl, depending on the NMDA dose. The protective efficacy of brimonidine in this chicken injury model was in a similar range as that seen in mammalian models [9]. The results show that the α2-ADR agonist brimonidine has neuroprotective effects on injured chicken RGCs. These data support the notion that α2-ADR agonists have protective effects on RGCs in a variety of retinal injuries caused by phototoxicity, excitotoxicity, ischemia, optic nerve crush and transection as well as by high intraocular pressure [6–9, 44] and that these neuroprotective effects are not restricted to the mammalian retina. The amount of brimonidine was similar to our previous experiments and it triggers an intracellular attenuation of the injury-response that comprises negative ERK-signaling feedback, leading to attenuated Müller cell dedifferentiation and gliosis [19]. The attenuation was seen by decreased P-ERK, expression of transitin and retinal progenitor cell genes was concomitant with a synergistic up-regulation of several negative ERK-signal feedback regulators including ERK-phosphatases, Raf1-, and growth factor receptor-binding proteins [19]. Thus, adrenergic stress-signals modulate the acute glial function in retina and α2-ADR agonists enhance the attenuation of the glial injury response in the chicken retina. The present results showed that a single bolus dose of the α2-ADR agonist brimonidine produced a long-term protection of RGCs against degeneration of RGC, triggered by the NMDA excitotoxic injury. The brimonidine treatment attenuates the injury-induced dedifferentiation and gliosis of Müller cells [19], as shown in E18 chicken retina and we suggest that the attenuation contributes to the protective effects.

The isodensity maps of the injured retinas showed that the loss of Brn3a+ RGC was higher in the dorsal retina than in the ventral one suggesting that dorsal RGCs are more sensitive to NMDA. The effect of brimonidine was therefore clearer in the dorsal retina than in the nasal (Fig 7). Our results showed that the ventro-temporal quadrant was less affected, which is consistent with previous data from chicken that report less effect of NMDA in the temporal aspect of the retina and eye [3, 45]. It remains to study if this regional difference reflects the vulnerability of RGC or the physiological properties of the retina for example in relation to the pecten oculi.

Brn3a has been used as a chicken RGC marker [28, 46–51]. Brn3a drives RGC differentiation and Brn3a+ nuclei are present in the GCL at E5-6 [28]. Retrograde labeling of RGCs from the optic nerve has been the golden standard method to identify RGCs. The use of Brn3a as a RGC marker has advantages: It is a non-invasive technique, it labels the majority RGCs, it can be combined with other markers and Brn3a is localized to the nucleus of the RGCs, which facilitates cell counting [24, 25].

Rager and Rager [32] estimated that there are in total 2.8x10^6^ RGCs in the P2 chicken retina, based on the number of optic nerve fibers. Extrapolation of counts of Nissl-stained cells in the GCL from sample areas and subtraction of displaced amacrine cells gave estimations of 4.8x10^6^ RGCs in P8 retina (Budnik et al., 1984, Chen et al., 1999). We found consistently in total 1.7–2.0x10^6^ Brn3a+ cells in the E12 to P11 retina (P11, 1,99x10^6^ ± 5,5x10^4^ RGCs) using the flat-mount retina and automated counting method. We did not see any increase or decrease in the total number of Brn3a+ cells after E12. Previous RGC number estimations report higher numbers in the embryonic retina than after hatching [33]. A constant number of RGCs after E12 is consistent with the notion that genesis and naturally occurring death of RGCs are both over by E12 [52]. The method may underestimate the number of RGC because not all RGC may express Brn3a and because RGC with low levels of Brn3a may be classified as negative and therefore be missed when using the automatic counting. In rodent retinas, Brn3a+ labeling was expressed in 92% of all retrograde tracing-identified RGCs leaving aside melanopsin RGCs and half of the ipsilateral projection [40]. Assuming a similar efficacy in chicken, then the total number of RGCs should be increased with 8%. Even though the algorithm is designed to compensate for underestimation of cell number if cells are over-lapping the relatively higher density of RGCs in the chicken retina compared to rodents may infer a lower density. We therefore manually calculated Brn3a+ and DAPI stained nuclei in the E18 GCL (Fig 2) and the average density of Brn3a cells in the sample areas was 11.0x10^3^±2.8x10^3^ cells/mm^2^ compared to 10.1x10^3^±1,2 x10^3^ cells/mm^2^ when automatically counted. The difference is 9.2% and may indicate that the automated cell counting underestimates the cell number and density. However, the sample areas were not from the peripheral rim of the retina or pecten, which has a lower RGC density. The automatic counting included these regions.

The manual counting showed that 72% of all DAPI-stained nuclei in the GCL of E18 chicken retina were Brn3a positive, indicating that 28% of the cells in the GCL were most likely Brn3a negative RGC or displaced amacrine cells. This fraction is similar to previous data on displaced amacrine cells that accounted for 20–30% of the cells in the GCL [53, 54].

Our data give an average number of 1.8x10^6^ Brn3a+ RGCs and a density of 9.3x10^3^ cells/mm^2^ in the post-hatch chicken retina. Previous studies report 4,5x10^6^ cells with the average density of 10.0x10^3^ cells/mm^2^. This discrepancy may be explained because Chen and co-workers described regions with increased RGC density [31] while the present study does not. The number of RGCs may also vary depending on the strain of chicken. The computer generated Brn3a+ RGC isodensity maps indicated that cells were distributed rather homogeneously across the central regions of the retina at developmental ages from E8 to P11. The exception was the peripheral rim and the region of the pecten oculli. The average number and cell density in quadrants and halves of the retina, compensated for the pecten oculli area, support an even RGC density. Interestingly in Chen et al. (2004), the total number of cells was deduced from the density of sample regions that was determined by subtraction of non-RGCs. Such non-RGCs were defined as cells resistant to retrograde degeneration after optic nerve injury [31]. Our data indicate a regional differential susceptibility to injury by RGC and that may have influenced previous RGC density estimations.

In conclusion, the automated Brn3a+ RGC counting method, developed for the rodent retina [40], was used to analyze the chicken retina. The number, density and distribution of Brn3a+ RGCs in normal embryonic and post-hatch, as well as in excitotoxically injured chicken retina were analyzed. No high-density areas were found in the retina, in contrast to previous data, however, a dorsal region of the retina showed increased susceptibility to the excitotoxic injury. We also conclude that pretreatment with the α2-ADR agonist brimonidine protected RGCs against the injury in the chicken retina in a fashion that is similar to the neuroprotection that has been seen in mammals.

## Supporting Information

S1 Fig Micrographs of Brn3a+ RGC in flat-mount retina 7 days after NMDA administration with BMD pretreatment and control. (PDF)

S2 Fig Micrographs of Brn3a+ RGC in flat-mount retina 14 days after NMDA administration with BMD pretreatment and control. (PDF)

S1 Table Number of Brn3a+ RGC, retinal area and cell density in each quadrants of embryonic and post-natal control retina. (PDF)
