# Supplementary material for: Neuroprotection by α2-Adrenergic Receptor Stimulation after Excitotoxic Retinal Injury: A Study of the Total Population of Retinal Ganglion Cells and Their Distribution in the Chicken Retina
Source: PLoS One. 2016 Sep 9;11(9):e0161862. doi: 10.1371/journal.pone.0161862 (PMC5017579; doi:10.1371/journal.pone.0161862)
Supplement: S2 Fig — (PDF) [file pone.0161862.s002.pdf]

S2 Fig. Micrographs of Brn3a+ RGC in flat-mount retina 14 days after NMDA administration with BMD pretreatment and control.

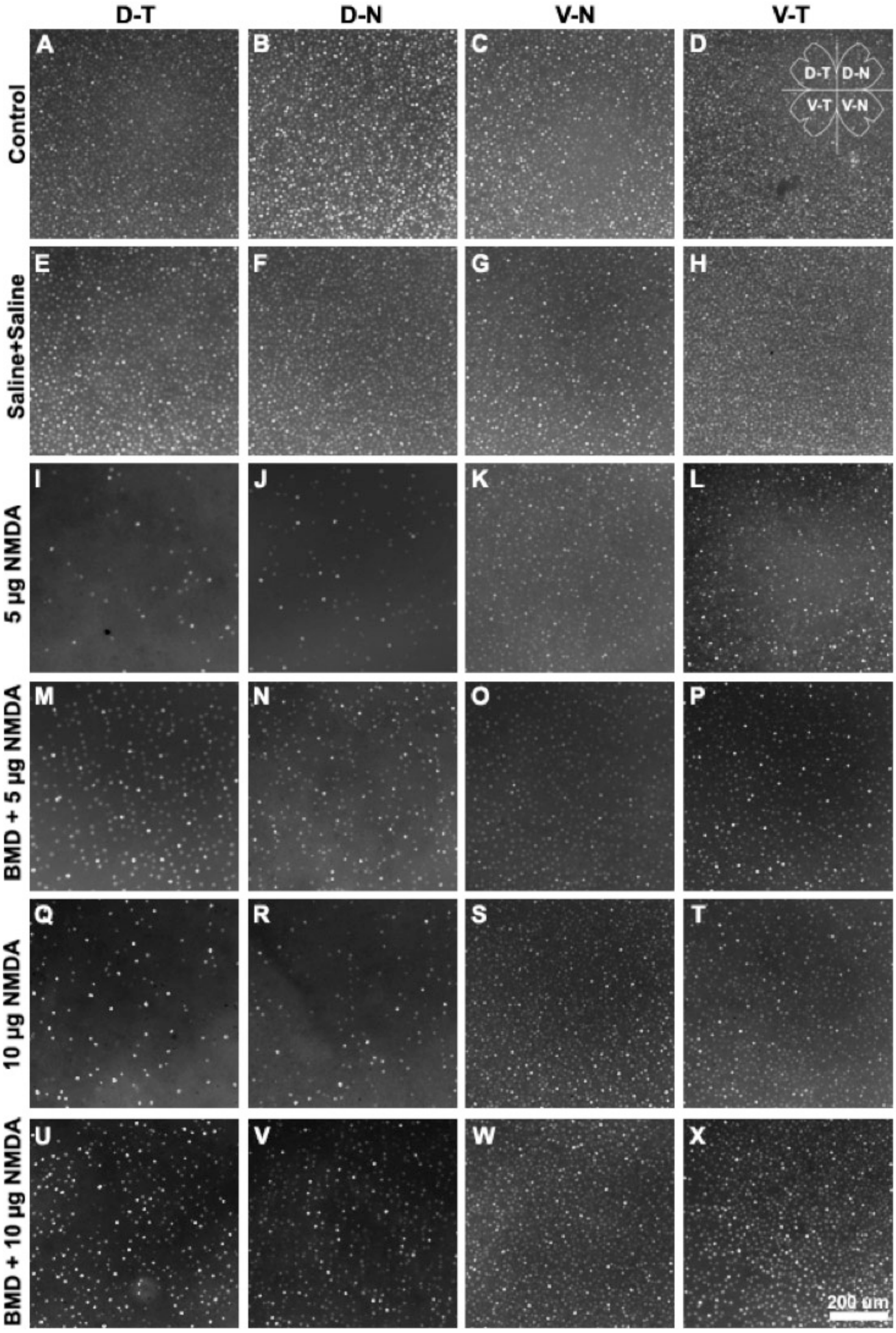

Representative fluorescence micrographs of Brn3a+ cells in the quadrants of flat-mounted retina at 14 days post-lesion. (A-D) Normal control retina with schematic diagram of the four retinal quadrants in panel D. Experimental groups were: (E-H) Injections of saline + saline, (I-L) saline + 5 μg NMDA, (M-P) brimonidine + 5 μg of NMDA, (Q-T) saline + 10 μg of NMDA, and (U-X) brimonidine + 10 μg of NMDA. BMD; brimonidine, D-T; dorso-temporal, D-N; dorso-nasal, V-N; ventro-nasal, V-T; ventro-temporal.
