# Supplementary material for: Neuroprotection by α2-Adrenergic Receptor Stimulation after Excitotoxic Retinal Injury: A Study of the Total Population of Retinal Ganglion Cells and Their Distribution in the Chicken Retina
Source: PLoS One. 2016 Sep 9;11(9):e0161862. doi: 10.1371/journal.pone.0161862 (PMC5017579; doi:10.1371/journal.pone.0161862)
Supplement: S1 Table — (PDF) [file pone.0161862.s003.pdf]

**S1 Table. Number of Brn3a+ RGC, retinal area and cell density in each quadrants of embryonic and post-natal control retina.**

| | | Retinal quadrants | | | |
|---|---|---|---|---|---|
| Age | | D-T | D-N | V-N | V-T |
| E8 | Brn3a$^{+}$RGCs | 376,197±42,555 | 359,002±36,347 | 368,912±40,159 | 362,185±42,115 |
| | Area[1] | 25.5±1.6 | 24.7±0.5 | 28.2±1.9 | 29.1±1.5 |
| | Density[2] | 14,782±1,534 | 14,575±1,693 | 13,109±1,419 | 12,518±1,884 |
| E9 | Brn3a$^{+}$RGCs | 350,266±18735 | 386,533±38,427 | 355,580±62,884 | 362,785±29,582 |
| | Area | 27.3±0.7 | 27.0±1.5 | 30.4±1.7 | 27.7±4.9 |
| | Density | 12,857±986 | 14,383±1,866 | 11,651±1,408 | 13,244±1,275 |
| E10 | Brn3a$^{+}$RGCs | 417,678±85,864 | 406,397±26,617 | 312,096±55,821 | 397,804±61,031 |
| | Area | 30.1±6.2 | 29.5±2.1 | 23.5±1.6 | 29.2±5.9 |
| | Density | 13,886±276 | 13,838±1,387 | 13,253±2,093 | 13,765±1,507 |
| E12 | Brn3a$^{+}$RGCs | 458,304±63,954 | 477,170±65,788 | 458,208±133,176 | 396,694±59,411 |
| | Area | 40.0±1.2 | 37.7±2.8 | 43.8±4.9 | 36.5±2.0 |
| | Density | 11,426±1,301 | 12,011±2,797 | 10,466±2,725 | 10,956±2,240 |
| E14 | Brn3a$^{+}$RGCs | 516,752±42,261 | 484,145±87,665 | 410,584±85,226 | 406,314±75,235 |
| | Area | 49.7±5.7 | 51.3±1.3 | 40.5±5.6 | 43.8±2.3 |
| | Density | 10,468±1,200 | 9,455±1,818 | 10,079±921 | 9,240±1,377 |
| E18 | Brn3a$^{+}$RGCs | 520,430±54,965 | 507,268±81,132 | 458,708±56,336 | 397,664±75,354 |
| | Area | 46.2±6.3 | 50.8±6.7 | 46.8±6.4 | 44.2±2.5 |
| | Density | 11,384±1,547 | 10,116±2,057 | 10,054±2,524 | 8,969±1,454 |
| E20 | Brn3a$^{+}$ RGCs | 445,645±25,425 | 522,656±86,249 | 405,036±52,241 | 444,814±75,755 |
| | Area | 48.3±0.2 | 59.3±2.0 | 40.4±3.8 | 50.3±6.1 |
| | Density | 9,234±560 | 8,812±1,339 | 10,112±1,832 | 9,051±2,629 |
| P4 | Brn3a$^{+}$RGCs | 464,000±35,309 | 461,607±50,097 | 495,463±35,862 | 405,481±39,926 |
| | Area | 49.6±0.7 | 47.9±2.6 | 55.2±3.5 | 44.8±5.2 |
| | Density | 9,361±850 | 9,625±685 | 8,974±400 | 9,221±2,105 |
| P11 | Brn3a$^{+}$RGCs | 543,942±95,632 | 502,619±72,362 | 468,080±13,827 | 479,357±9,399 |
| | Area | 52.6±1.4 | 55.7±4.3 | 53.7±5.2 | 50.2±9.9 |
| | Density | 10,329±1,685 | 9,050±1,434 | 8,756±614 | 9,805±1,979 |

The number of Brn3a$^{+}$RGCs was obtained individually for each quadrant. The mean density (Brn3a$^{+}$RGCs/mm$^{2}$) was calculated from the total number of Brn3a$^{+}$RGCs and the total retinal area. Numbers are Mean±SD.There were no significant differences between quadrants of each retina in all groups. D-T= Dorso-Temporal; D-N= Dorso-Nasal; V-N= Ventro-Nasal; V-T= Ventro-Temporal.
